# Catalyst-controlled functionalization of carboxylic acids by electrooxidation of self-assembled carboxyl monolayers

**DOI:** 10.1038/s41467-022-28992-4

**Published:** 2022-03-14

**Authors:** Heather A. Hintz, Christo S. Sevov

**Affiliations:** Department of Chemistry and Biochemistry, The Ohio State University, 151W Woodruff Avenue, Columbus, OH 43210 United States

**Keywords:** Electrocatalysis, Synthetic chemistry methodology, Surface assembly

## Abstract

While the electrooxidative activation of carboxylic acids is an attractive synthetic methodology, the resulting transformations are generally limited to either homocoupling or further oxidation followed by solvent capture. These reactions require extensive electrolysis at high potentials, which ultimately renders the methodology incompatible with metal catalysts that could possibly provide new and complementary product distributions. This work establishes a proof-of-concept for a rare and synthetically-underutilized strategy for selective electrooxidation of carboxylic acids in the presence of oxidatively-sensitive catalysts that control reaction selectivity. We leverage the formation of self-adsorbed monolayers of carboxylate substrates at the anode to promote selective oxidation of the adsorbed carboxylate over a more easily-oxidized catalyst. Consequently, reactions operate at lower potentials, greater faradaic efficiencies, and improved catalyst compatibility over conventional approaches, which enables reactions to be performed with inexpensive Fe complexes that catalyze selective radical additions to olefins.

## Abstract (web summary)

This work leverages substrate self-assembly at an electrode to promote selective substrate electrooxidation in the presence of oxidatively sensitive catalysts. This strategy is applied to decarboxylative coupling of carboxylic acids with olefins.

## Introduction

**Fig. 1:**
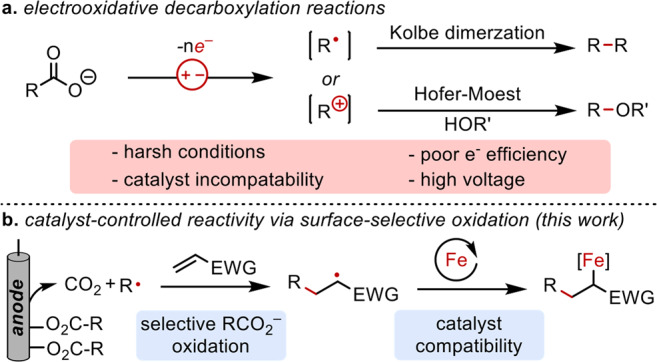
Electrochemical Decarboxylation in Synthesis. **a** Common electrooxidative decarboxylation reactions. **b** Selective oxidation of carboxylates enabled by dielectric formation on an anodic surface.

Decarboxylative functionalization reactions of alkyl carboxylic acids have long served as important methodologies in organic synthesis^1–3^. The ubiquity of such acids in bioavailable and commodity chemicals renders them attractive sources of reactive alkyl fragments as an alternative to alkyl halides^4,5^. Access to the carbon fragment requires the formation of a transient carboxyl radical, which can subsequently undergo rapid decarboxylation (>10^5^ s^−1^) to release CO_2_ and an alkyl radical^6^. While classical methodologies rely on stoichiometric chemical oxidants or synthetic derivatization to pre-activate the carboxyl fragment, electrooxidation of a carboxylate offers a direct route to the key carboxyl radical but with greater atom and step economy than the former methodologies^7–16^. However, despite the relatively low potentials necessary for carboxylate oxidation (~1.5 V vs Fc/Fc^+^), direct electrolysis of carboxylic acids often requires excessive oxidizing equivalents and cell potentials that exceed 20 V^17,18^. These continuous and extreme potentials present compatibility issues for catalysts that could capture and control the reactivity of transient alkyl radicals. In contrast, catalyst-controlled decarboxylative functionalization has been successfully demonstrated with photoredox methodologies^19–27^ but remains extremely underdeveloped under electrochemical control. Rather, products from electrooxidative decarboxylation reactions are limited to radical homocoupling that forms Kolbe dimers, Hofer–Moest solvent capture to form ethers, or intramolecular radical cyclizations (Fig. 1a)^28–34^.

We report an alternative strategy for electrooxidative activation of carboxylic acids that provides compatibility with metal catalysts to control the reactivity of the resulting carbon-centered radical (Fig. 1b). We leverage the known, but synthetically-underutilized, phenomenon of carboxyl aggregation at an anodic surface to form self-assembled monolayers (SAMs). The electrode-bound carboxylates undergo selective oxidation in preference to more easily-oxidized metal catalysts, substrates, and anions^35–40^. Critically, the SAM serves as a barrier that prevents diffusion of oxidizable compounds to the anodic surface but does not irreversibly passivate the electrode^41^. The assembled layer also prevents oxidative leaching of metals, which allows electrolysis to be performed with inexpensive anodes like Ni in place of corrosion-resistant electrodes like Pt or boron-doped diamond.

Electrochemical reactions at insulating layers on electrode surfaces are counterintuitive, but the approach has been shown to offer a unique selectivity for the redox of compounds within the surface layer over redox of homogeneous species that cannot readily diffuse into the bilayer^41^. This control over Faradaic reactions is particularly useful when the desired redox reaction is of a compound that is more difficult to oxidize/reduce than another compound in solution. Chung and coworkers have demonstrated the effect of insulating SiO_2_ layers on a silicon electrode for the selective reduction of protons to hydrogen atoms that subsequently mediate chemical reactions at the SiO_2_-solution interface^42,43^. Despite the potential of this powerful strategy for electrosynthesis, the field remains highly underdeveloped. To the best of our knowledge, the only known reactions employing this strategy include one report of H-atom abstraction of C–H bonds and two reports of metal-ion reduction to form nanoparticles^42–45^.

**Fig. 2:**
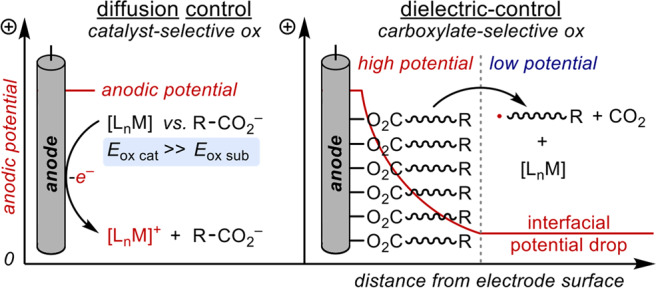
Interfacial Potential. Interfacial potential drop at an anode with a self-assembled double layer of carboxylates.

With these insights, we envisioned that carboxylate assembly at an electrode surface could similarly create a dielectric layer that promotes selective oxidation of carboxylates over readily-oxidized catalysts. Illustrated in Fig. 2, diffusion of the catalyst to the high-potential regime near the electrode surface is blocked. This strategy is implemented in a rare example of a metal-catalyzed decarboxylative functionalization of carboxylic acids under electrochemical conditions. Specifically, we demonstrate the Fe-catalyzed decarboxylative alkylation of electron-deficient alkenes with unactivated carboxylic acids. Mechanistic studies including electrochemical impedance spectroscopy (EIS) highlight the importance of substrate adsorption at the anode to protect the Fe catalyst from oxidative degradation. Finally, this methodology represents one of only a handful of examples of chemical synthesis at a dielectric layer.

## Results

### Electrooxidative decarboxylation of carboxyl SAMs

Our initial efforts towards catalyst-controlled decarboxylative functionalization centered on identifying conditions that enable electrooxidation of carboxylic acids with good faradaic efficiencies (>25%) at lower potentials than classic conditions for Kolbe electrolysis (Table 1). These conditions would be later extended to catalyst-controlled protocols. The efficacies of the preliminary conditions for oxidative homocoupling were benchmarked by the extent of formation of product **1**. Decanoic acid was selected as the model substrate because products and byproducts are easily monitored by GC, and the acid contains a long alkyl chain that has been previously reported to promote SAM formation at anodes^35,38^. Electrolysis was performed with an RVC electrode at a constant cell potential of 6 V until 4 F/mol had passed. Reactions under conventional Kolbe conditions of methanol and KOH as the base exhibited high currents (>10 mA) but almost none of the decanoic acid was consumed even after prolonged electrolysis beyond 4e- equivalents (entry 1). This result highlights the challenge of bypassing parasitic oxidation of reagents (hydroxide, methoxide, methanol) in favor of the carboxylate at low applied potentials.

**Table 1:** Identification of mild conditions for Kolbe homocoupling.


Entry	Deviation from standard	% yield
1	(±) RVC, 20 mol% KOH in MeOH	5
2	(±) RVC, 100 mM KPF_6_ added	0
3	(±) RVC, 100 mM TBAClO_4_ added	0
4	(±) RVC, 100 mM TBAPF_6_ added	0
5	(±) RVC, 20 mol% piperidine	42
6	(±) RVC, 20 mol% lutidine	38
7	(±) RVC	31
8	(±) RVC, pulse 10 V at 10 Hz	61
9	(±) RVC, pulse 10 V at 94 Hz	67
10	(±) Ni foam	56
**11**	**none**	**71**
12	(±) Pt	3
13	(±) Pt/RVC	14
14	3 V instead of 6 V	0
15	4.9 V instead of 6 V	64
16	no e-chem	0
17	(±) RVC, 10 mol% Ni(OAc)_2_•4H_2_O	18

We replaced oxidatively-sensitive solvents and bases with acetonitrile and a supporting electrolyte, but conversion remained low (<5%) and no product was detected (entries 2–4). We hypothesized that anions from exogenous supporting electrolytes disrupt the targeted formation of a carboxyl layer at the anodic surface^46^. As such, we next evaluated amine and pyridine bases that could deprotonate the acid to form an ammonium carboxylate salt to serve as the supporting electrolytes^47^. The resulting reactions were the first to form significant quantities of product: up to 42% (entries 5–7). We discovered that pulsed electrolysis could further improve the yield of **1**. Various pulse frequencies, duty cycles (time on vs. time off), and pre-polarizations were programmed into the experiments with the aim of promoting carboxylate aggregation at the anode and selective oxidation of the substrate (entries 8–9). While pulsing doubled yields from reactions with RVC anodes, we discovered that reactions performed with Ni-foam anodes in place of RVC generated products in equal or better yield at a constant voltage (entry 11). Surprisingly, replacing Ni with Pt electrodes caused an extremely high cell resistance under the non-Kolbe conditions and resulted in low yields (entry 12). Finally, control experiments confirmed that electrochemistry is required (entry 15) and that solvated Ni, which may be generated by oxidative corrosion of the Ni anode, is not responsible for the observed reactivity (entry 16). These conditions for electrooxidative decarboxylation of primary carboxylic acids are among the mildest reported to date.

### Mechanistic investigation

**Fig. 3:**
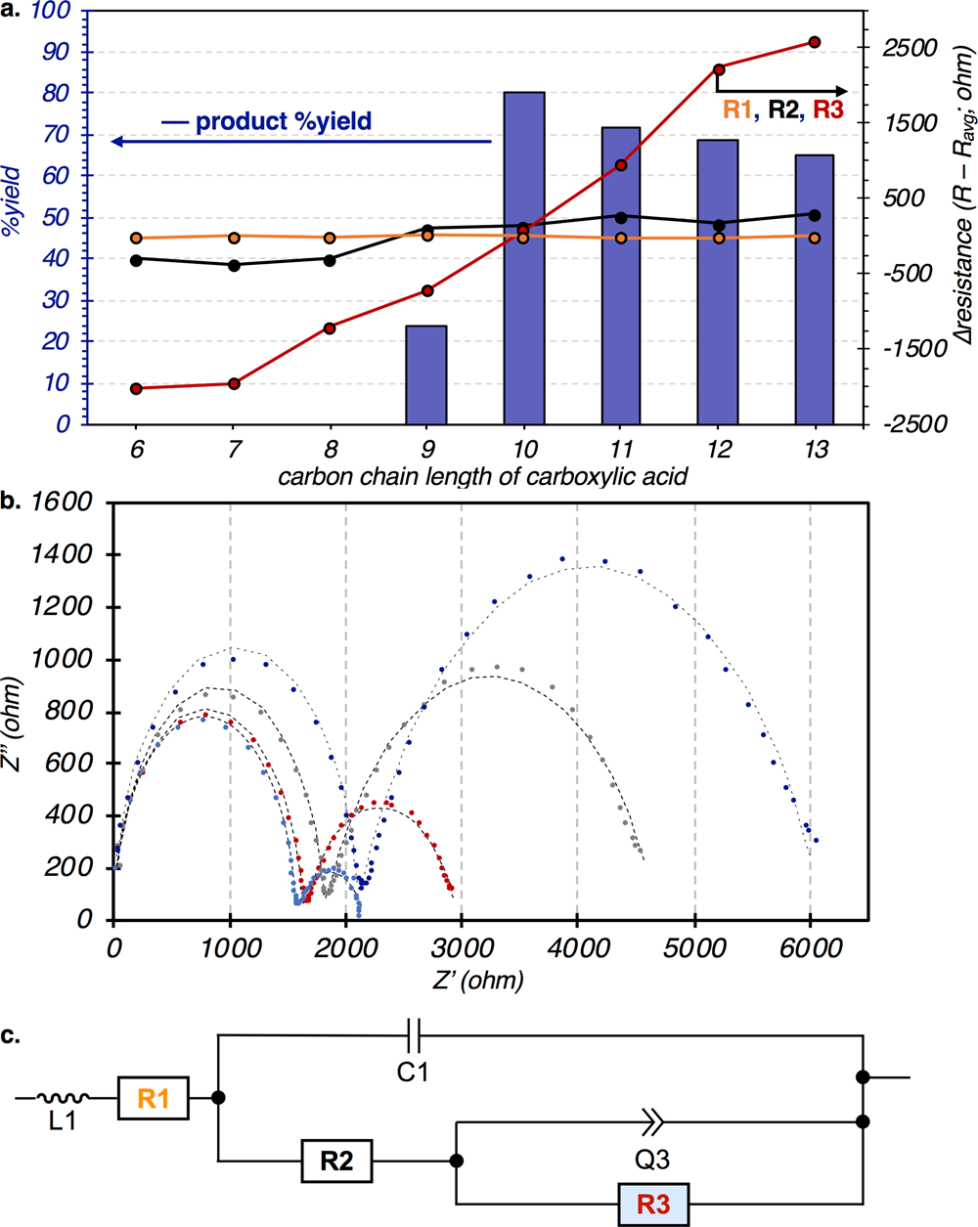
Structure-Reactivity Studies of Carboxylic Acids. **a** Comparison of the yield of **1** (left axis, bars) and independent resistors from EIS fittings (right axis, trace) as a function of varying chain lengths. **b**. Nyquist plots from EIS and modeled fit (dashed traces) of C6, C8, C10, and C12 after 30 min of electrolysis. **c** Illustration of the equivalent circuit employed to fit the EIS data.

The role of the anodic material and the importance of how electrochemical potential is applied led us to investigate the chemistry at the electrode interface. The mild conditions for Kolbe decarboxylation could then be translated to catalyst-controlled methodologies with selectivities beyond simple homocoupling of alkyl radicals. This investigation was particularly important following our discovery that the yields of homocoupled products correlate with the chain length of the carboxylic acid. While small changes to the length of an alkyl fragment typically have little impact on the outcomes of homogeneous reactions, electrolysis of octanoic acid (C8) formed trace quantities of the product while those with decanoic acid (C10) formed product in 71% yield (Fig. 3a, blue trace). We investigated the chemistry of the interfacial layer at the electrode using electrochemical impedance spectroscopy under the standard conditions with both Ni-foam and RVC anodes to best replicate the reaction conditions. EIS measurements were acquired at an applied voltage of 2 V with an overlaid voltage perturbation of ±200 mV from a high frequency of 1 MHz to a low frequency of 0.5 Hz. These analyses were performed for acids ranging in length from hexanoic (C6) to tridecanoic (C13) acid both at the beginning of the reaction with a fresh electrode and after 30 min of electrolysis.

EIS measurements with pristine electrodes are nearly identical for all carboxylic acids prior to electrolysis, and the spectra could be fitted to a simple Randles circuit (Supplementary Fig. 6)^40^. However, EIS measurements performed after 30 min of electrolysis with the varying carboxylic acids resulted in dramatically-different impedance spectra. No further change to the spectra was observed following evenlonger periods of electrolysis (6 h). The overlaid Nyquist plots reproduced in Fig. 3b reveal nearly identical semicircles at high frequencies that match those of the pristine electrode. However, the low-frequency regime of the long-chain acids exhibits a finite-length Warburg element that is consistent with the formation of a surface layer on the electrode^40,48–50^. These data can be modeled by an equivalent circuit that decouples the Warburg element into a parallel Q factor (Q3) and resistor (R3)^48,51^, and the fitted traces are illustrated as dashed lines in Fig. 3b.

Of all terms in the circuit, R3 was found to have the greatest variation as a function of acid length. This variation in R3 among acids is best illustrated on the secondary Y-axis of Fig. 3b as a difference of the measured R3 of each acid from to the average resistance (R3_avg_) across all acids. The differences in resistance due to the electrolyte (R1, orange trace) or electron transfer (R2, black trace) as a function of chain length are negligible. In contrast, R3 (red trace) is largest with long-chain acids and smallest with short-chain acids, spanning a range of nearly 5000 ohms. This term dominates the low-frequency regime where Faradaic redox occurs and is typically attributed to resistance to diffusion to the electrode. The increased resistance caused by long-chain carboxylates is consistent with the formation of stable SAMs that are supported by strong dispersion interactions between the long aliphatic groups^38^. Surface layers of carboxylate substrates with the longest hydrocarbon chains would suppress diffusion to the electrode surface the most. Additionally, studies revealed that the formation of carboxylate monolayers is reversible. Anodes polarized in solutions of long-chain acids (C12) to form stable SAMs were then placed into solutions of short-chain acids (C6). EIS measurements taken immediately after the exchange displayed high resistance, which is consistent with a SAM of the C12 acid of the first solution. However, the impedance spectra rapidly changed, and the resistance matched that of an anode polarized in a solution of just the C6 carboxylic acid (Fig. 3b, light blue trace). These experiments suggest that the increased resistance is not the result of irreversible electrode fouling and that the interface is dynamic and undergoes carboxylate exchange. (Supplementary Fig. 15)

**Fig. 4:**
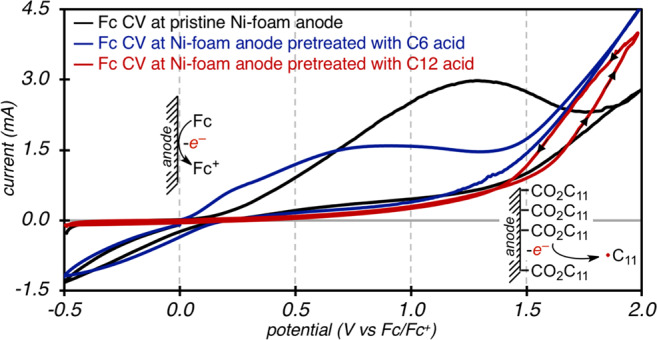
CV Studies of SAMs. CV of ferrocene (10 mM) with pristine Ni-foam (black), C6 pretreated Ni-foam (blue), and C12 pretreated Ni-foam (red). CV conditions: 0.1 M KPF_6_ in MeCN, 100 mV/s scan rate, room temperature, Ni-foam working electrode (WE), and RVC counter electrode (CE).

We next evaluated the effect of carboxyl SAM formation on the Faradaic electron transfer between ferrocene and the semi-insulated anode^52^. With ferrocene (Fc) serving as a redox probe, cyclic voltammograms (CVs) were measured under the standard reaction conditions by sweeping from −0.5 to +2.0 V at various Ni-foam working electrodes (WEs). Summarized in Fig. 4, CVs were measured with (i) a pristine Ni-foam WE, (ii) a Ni-foam WE that was polarized in a solution of a long-chain carboxylic acid (C12), and (iii) a Ni-foam WE that was polarized in a solution of a short-chain carboxylic acid (C6). CVs of Fc with a pristine Ni-foam WE reveal an expected onset current at +0.1 V that results from oxidation of Fc at a high surface-area anode (black trace). In contrast, this oxidation event was completely suppressed when the identical measurement was performed using a Ni-foam anode pretreated with a long-chain carboxylate (red trace). Rather, an oxidative current was only observed at high potentials of +1.3 V, which is attributed to carboxylate oxidation.

The CV experiments suggest that self-assembly of the long-chain carboxylate generates a dielectric layer that inhibits diffusion and subsequent redox of the Fc probe but still allows for oxidation of the adsorbed carboxyl substrate. Redox within the adsorbed layer is supported by the increased current response when the CV scan is reversed from +2 V, which results in crossing traces (Fig. 4, red trace)^42^. As a result, the selectivity for oxidation is strongly dependent on the length of the carboxylic acid. Specifically, the oxidation of Fc is detected at the expected potential when CVs are measured with Ni anodes that were pretreated in hexanoic acid (C6, blue trace). These data are consistent with the EIS measurements of increased resistance to diffusion (R3) as the chain length of the carboxylic acid increases. Most importantly, these findings establish a strategy to perform selective oxidation of carboxylates at high potentials even in the presence of redox-active compounds that are more easily oxidized.

## Discussion

Having identified conditions that promote the formation of carboxyl SAMs at the anode, we leveraged the selective electrooxidation of the adsorbed substrate to effect catalyst-controlled decarboxylative functionalization of carboxylic acids with electron-deficient alkenes. Such Giese-type additions of transient alkyl radicals into olefins have attracted significant interest in recent years but require pre-activation of the carboxylate to allow for decarboxylation under mild conditions that are compatible with catalysts^53–58^. The necessity for catalyst control is clearly exemplified by entry 1 in Table 2, where direct electrolysis of decanoic acid in the presence of ethyl acrylate generates a complex mixture of products (**2**–**5**).

**Table 2:** Conditions for metal-catalyzed radical additions.


Entry	Deviation from standard	% yield	ratio 2: (3 + 4 + 5)
1	no catalyst	76	0.2: 1
2	5 mol% Co(acac)_3_	0	–
3	5 mol% Cr(acac)_3_	0	–
4	5 mol% Cu(OAc)_2_	0	–
5	5 mol% Mn(OAc)_2_(H_2_O)_4_	19	0.3: 1
6	5 mol% Ni(OAc)_2_(H_2_O)_4_	22	0.8: 1
7	5 mol% Fe(OAc)_2_	67	6: 1
**8**	**none (5** **mol% Fe(acac)**_**3**_**, 4.2** **V)**	**80**	**6: 1**
9	10 mol% Fe(acac)_3_	56	18: 1
10	1.1 equiv Fe(acac)_3_, no e-chem	0	–
11	3 V instead of 4.2 V	5	>50: 1
12	5 V instead of 4.2 V	79	3: 1
13	7 V instead of 4.2 V	83	1: 1

We evaluated a wide range of transition metal salts and complexes that could capture alkyl radicals generated by oxidative decarboxylation to direct product selectivity. Reactions with catalytic quantities of Co, Cr, Cu, Mn, and Ni-based salts all inhibited decarboxylation of dodecanoic acid under the standard conditions (entries 2–6). Analysis of these low-yielding reactions revealed a low diffusion resistance and a high current, which implies that the added salts can disrupt the self-assembly of carboxylate substrate at the anode that is necessary for selective oxidation. These metrics were used as an assay to rapidly pre-screen a wide range of complexes as potential catalysts. Solutions with Fe-based salts were most promising, as EIS measurements suggested that the desired carboxyl SAM was retained. Translation of this simple analysis to the reaction scale proved highly effective. Reactions conducted with 5 mol% Fe(OAc)_2_ or Fe(acac)_3_ (entries 7 and 8) dramatically improved the selectivity for the desired addition product **2** over other products generated during catalyst-free electrolysis. Iron-catalyzed radical additions to olefins have been previously reported but such catalysts are incompatible with typical conditions for electrooxidative decarboxylation^13,59,60^.

**Fig. 5:**
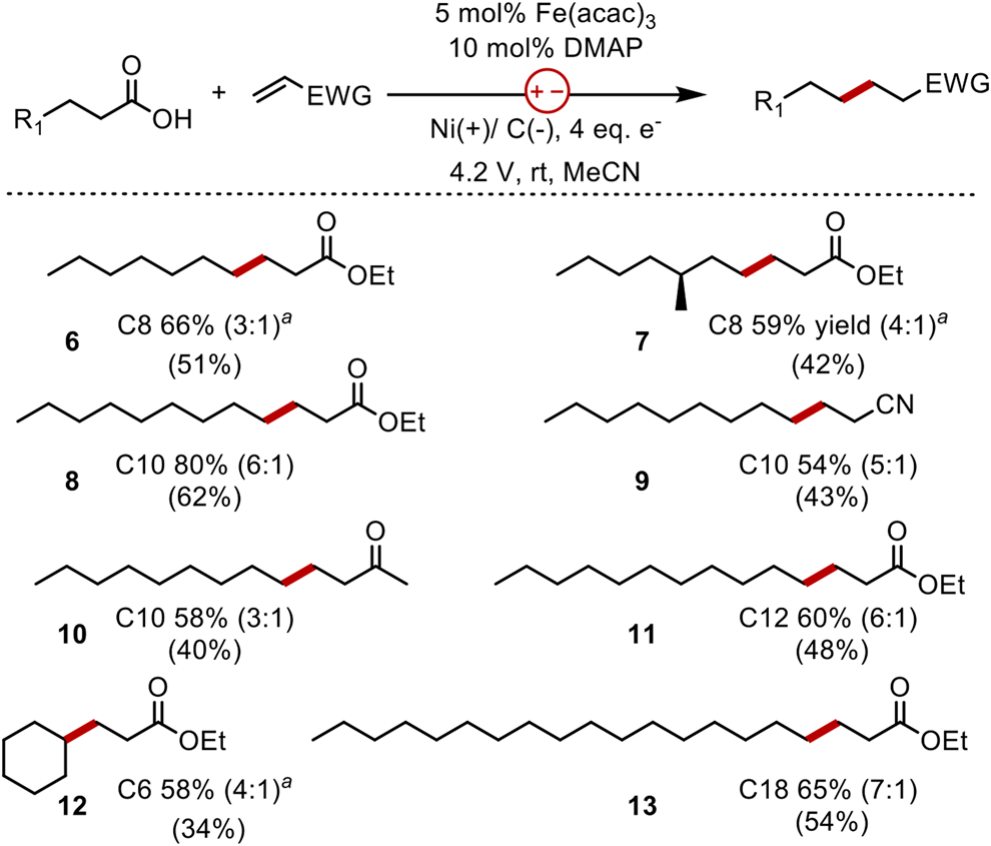
Substrate scope. Scope of electrochemical decarboxylation. The ratio represents the yield of the addition product to the sum of all other isomers. Isolated yields are reported in parenthesis. ^a^RVC used in place of Ni foam.

Selectivity for the additional product can be further increased with increased loadings of Fe(acac)_3_, albeit at the expense of yield (entry 9). Control studies in the absence of electrolysis with stoichiometric quantities of Fe(acac)_3_ reveal that electrolysis is necessary and that Fe^III^ alone is not responsible for decarboxylative activation of the substrate (entry 10). The applied voltage similarly impacts selectivity and yield, where reactions performed at low voltages are more selective than high voltage reactions but are lower yielding (entries 11–13). As a result, reactions conducted at 4.2 V with 5 mol% Fe(acac)_3_ provided an ideal balance of product selectivity and yield. To further demonstrate the proof-of-concept, we applied this strategy to the decarboxylative addition of a range of carboxylic acids to electron-deficient olefins (Fig. 5). A simple evaluation of the reaction mixture by EIS prior to electrolysis proved to be an excellent predictor of reaction outcome because it revealed whether the carboxylic acid creates a SAM (Supplementary Fig. 18). This methodology is particularly applicable to the modification of fatty acids that readily form SAMs. Products from these reactions are formed selectively in good yield and can be isolated from the minor byproducts in slightly lower yields. Mechanistic studies reveal that reductive degradation of the electron-deficient alkenes limits product formation under standard conditions, but yields can be increased by an additional 15% with the second addition of acrylate midway through the reaction.

**Fig. 6:**
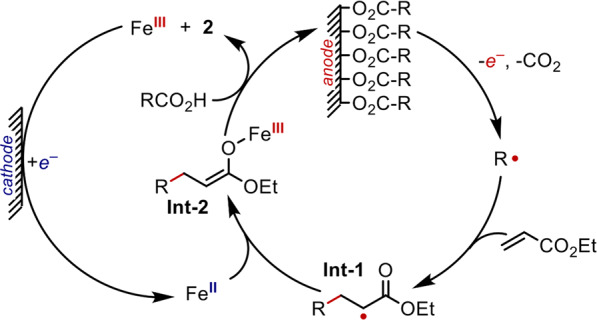
Proposed mechanism. Illustration of a possible mechanism supported by the data that involves redox at electrodes.

The formation of the observed products under metal-catalyzed conditions can be rationalized by the mechanism proposed in Fig. 6. Aggregation of the carboxylate anion at the polarized anode blocks preferential oxidation of the Fe catalyst. Instead, only the carboxylate is oxidized within this double layer. This dielectric-controlled oxidation of carboxylates over the Fe catalyst is further supported through EIS studies performed over the course of reactions with short-chain and long-chain acids. As expected, long-chain acids form a resistive layer while short-chain acids fail to establish a SAM that protects the catalyst from overoxidation (Supplementary Fig. 16). Additionally, reactions of a C10 carboxylic acid were performed with a pretreated Ni-anode containing a SAM of a C12 carboxylic acid. This competition experiment probes whether substrate activation occurs directly from the SAM, which we could determine by monitoring the time-dependent formation of products from the C12 substrate on the electrode versus the C10 substrate in solution (Supplementary Fig. 22). Despite the excess of the C10 acid, C12 products were formed in a 1:1 ratio with the C10 product in the first minutes of electrolysis. After this initial period, C10 products were formed predominantly, which suggests that the surface layer of C12 was consumed or exchanged with C10 in solution. Following selective oxidation of the surface-adsorbed carboxylate, the carboxyl radical undergoes rapid decarboxylation to reveal a nucleophilic alkyl radical that is captured by an electrophilic alkene. The unstable radical alpha to the carbonyl of **Int-1** couples with Fe^II^(acac)_2_ to form the Fe^III^(enolate) **Int-2**, which is easily protonated by another carboxylic acid to generate a product. Finally, Fe(III) is reduced at the cathode to regenerate the oxidatively-sensitive Fe(II) intermediate that is critical for controlling the product selectivity of the reaction. The proposed mechanism is consistent with a redox-neutral reaction that necessitates paired electrolysis at both the anode and cathode to form the addition product selectively. When the electrodes are separated in a divided cell, the product mixtures of the resulting reactions are similar to those of uncatalyzed reactions that preferentially form compounds **3**–**5** (Supplementary Fig. 17).

In summary, we report a rare and synthetically-underutilized strategy for electrooxidation of challenging substrates in the presence of oxidatively-sensitive catalysts that control reaction selectivity. Aided by mechanistic investigations, we have identified conditions that generate SAMs of carboxylate substrates at the anode to promote selective oxidation of the adsorbed substrate over a more easily-oxidized catalyst. Subsequent decarboxylation generates a carbon-centered radical that couples with olefins under the control of an inexpensive Fe catalyst to generate additional products. In contrast to the harsh conditions of the traditional Kolbe decarboxylation that preclude the use of catalysts, these reactions operate with lower potentials, greater faradaic efficiencies, and catalyst compatibility. Ongoing work aims to further leverage these SAM formations to enable reactions with a broad range of carboxylic acids.

## Methods

### General procedure for Kolbe homocoupling

In a nitrogen-filled glovebox, a 12 mL reaction vial was charged with a stir bar, decanoic acid (129 mg, 0.750 mmol), 4-dimethylaminopyridine (9 mg, 70 µmol), and MeCN (3 mL). The reaction vial was sealed with a septa-lined cap. Copper wire leads attached to a Ni foam electrode (6 mm × 30 mm) and an RVC electrode were pierced through the septa. The electrodes were submerged to a depth of 5 mm into the solution. The reaction was removed from the glovebox placed under N_2_. A cell potential of 6 V was applied at room temperature for 4 equiv. e- and the solution was vigorously stirred (700 rpm). Following electrolysis, a basic workup was performed with a 1 M aqueous solution of NaOH. Following extraction with hexanes, the mixture was concentrated and loaded onto silica. The resulting residue was purified by flash column chromatography. The conditions for chromatography and other data that are specific to each compound are given in the SI.

### General procedure for metal-catalyzed decarboxylative addition reactions

In a nitrogen-filled glovebox, a 12 mL reaction vial was charged with a stir bar, decanoic acid (129 mg, 0.750 mmol), MeCN (3 mL), 4-dimethylaminopyridine (9 mg, 70 µmol), Fe(acac)_3_ (13 mg, 38 µmol), ethyl acrylate (150 mg, 1.50 mmol), and MeCN (3 mL). The reaction vial was sealed with a septa-lined cap. A Ni-foam electrode (6 mm × 30 mm) and an RVC electrode (6 mm × 30 mm) were pierced through the septa and were submerged to a depth of 5 mm into the solution. The reaction was removed from the glovebox placed under N_2_. An oxidative current was then applied to the Ni-foam electrode (4.2 V, 4 equiv e-) at 25 °C and vigorously stirred (700 rpm). Following extraction with hexanes, the mixture was concentrated and loaded onto silica. The resulting residue was purified by flash column chromatography. The conditions for chromatography and other data that are specific to each compound are given in the SI.

### Cyclic voltammetry and electrochemical impedance spectroscopy (EIS)

Cyclic voltammetry and electrochemical impedance spectroscopy (EIS) was performed with a Biologic VSP multichannel potentiostat/galvanostat. Cyclic voltammetry was carried out in a three-electrode electrochemical cell, consisting of a glassy carbon disk working electrode (0.07 cm^2^, BASi), an Ag/Ag^+^ quasi-reference electrode (BASi) with 0.01 M AgBF_4_ (Sigma) in MeCN, and a platinum wire counter electrode (23 cm, ALS). The glassy carbon disk electrode was polished in a nitrogen-filled glovebox using diamond polish (15 µm, BASi) and anhydrous MeCN. All experiments were performed at a scan rate of 100 mV/s in a MeCN electrolyte containing 0.1 M KPF_6_ unless otherwise noted. Reference electrodes were calibrated against an internal voltage reference of ferrocene (1–10 mM). Reactions were conducted as two-electrode cells with a LANHE LAND battery testing system using nickel foam (1.5 mm × 250 mm × 200 mm, 110 ppi, 99.8% purity, purchased from Amazon.com) and RVC electrodes (purchased from ERG Aerospace).

## Supplementary information

Supplementary Information

## Data Availability

The data supporting the findings of the study are available within the paper and its Supplementary Information. Source data are provided with this paper.

## Source data

Source Data
