# Supplementary material for: Catalyst-controlled functionalization of carboxylic acids by electrooxidation of self-assembled carboxyl monolayers
Source: Nat Commun. 2022 Mar 14;13:1319. doi: 10.1038/s41467-022-28992-4 (PMC8921278; doi:10.1038/s41467-022-28992-4)
Supplement: Supplementary file 1 — Supplementary Information [file 41467_2022_28992_MOESM1_ESM.pdf]

Supplementary Information

Catalyst-Controlled Functionalization of Carboxylic Acids by Electrooxidation of Self-Assembled Carboxyl Monolayers

Heather A. Hintz, Christo S. Sevov*

Department of Chemistry and Biochemistry, The Ohio State University, 151 W Woodruff Avenue, Columbus, OH 43210, United States

sevov.1@osu.edu

Table of Contents

General Remarks……………………………………..……………….……..……………………S3
General Procedure for Kolbe Homocoupling………..……………..…..………………………………S3
General Procedure for Metal-Catalyzed Radical Addition…………..……………………………..S4
Additional Conditions Testes for Kolbe Homocoupling……………………………………..………..S5
List of metals Tested……………………………………...……………………………………………...S6
Procedure for Electrode Preparation…………………..………………………………………………S7
Procedure for Electrochemical Impedance Spectroscopy (EIS)……………………………..…….......S9
Pre- and post- electrolysis trials..………………………………..……………………………..…S10
Procedure for Divided Cell Reaction………………………………………………………………….S15
Substrate Scope Limitations…………………………………………………………………...…S16
Additional Mechanistic Experiments…………………………………………………………….S18
ElectraSyn Experiment……………………………………………………………………………...S19
Isolated Product Characterization………………………………………………………………….S19
References……………………………………………..……………………………………………S21
Reproduced Copies of Spectra……………………………………………………………………....S23

General Remarks

Unless otherwise noted, all experiments were conducted under a dry atmosphere of nitrogen to confirm that electrochemistry, rather than oxygen, is the source of oxidative turnover. Reaction cells were assembled in a nitrogen filled dry box, and all chemicals were used without further purification. Anhydrous acetonitrile was purchased from Millipore Sigma.

^{1}H NMR spectra were obtained at 400 or 600 MHz and chemical shifts were recorded relative to $CHCl_3$ in $CDCl_3$ (δ7.26 ppm). ^{13}C NMR were obtained at 101 MHz. ^{19}F NMR were obtained at 377 MHz. Proof of purity is demonstrated by copies of NMR spectra. NMR multiplicities are reported as follows: singlet (s), doublet (d), triplet (t), quartet (q), multiplet (m), broad signal (br). GC analysis was performed on an Agilent 7890B GC equipped with an HP-5 column (30 m x 0.32 mm x 0.25 µm film) and an FID detector. Quantitative GC analysis was performed by adding dodecane as an internal standard to the reaction mixture upon completion of the reaction. Response factors for the products relative to the internal standard were measured for reaction development.

All electrochemical analyses were carried out in a nitrogen-filled glovebox. Cyclic voltammetry and Electrochemical Impedance Spectroscopy (EIS) were performed with a Biologic VSP multichannel potentiostat/galvanostat. Cyclic voltammetry was carried out in a three electrode electrochemical cell, consisting of a glassy carbon disk working electrode (0.07 cm_2 , BASi), a Ag/Ag^+ quasi-reference electrode (BASi) with 0.01 M $AgBF_4$ (Sigma) in MeCN, and a platinum wire counter electrode (23 cm, ALS). The glassy carbon disk electrode was polished in a nitrogen-filled glovebox using diamond polish (15 µm, BASi) and anhydrous MeCN. All experiments were performed at a scan rate of 100 mV/s in a MeCN electrolyte containing 0.1 M KPF_6 unless otherwise noted. Reference electrodes were calibrated against an internal voltage reference of ferrocene (1-10 mM). Reactions were conducted as two-electrode cells with a LANHE LAND battery testing system using nickel foam (1.5 mm x 250 mm x 200 mm, 110 ppi, 99.8% purity, purchased from Amazon.com) and RVC electrodes (purchased from ERG Aerospace). Reactions were conducted in Fisherbrand disposable borosilicate glass tubes with a threaded end (16 x 100 mm).

General procedure for Kolbe dimerization as applied towards the synthesis of 1

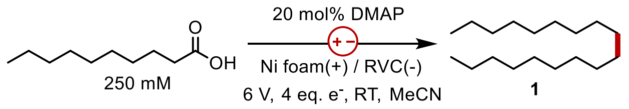

In a nitrogen filled glove box, a 12 mL reaction vial was charged with a stir bar, decanoic acid (129 mg, 0.750 mmol), 4-dimethylaminopyridine (9 mg, 70 µmol), and MeCN (3 mL). The reaction vial was sealed with a septa-lined cap. Copper wire leads attached to a Ni foam electrode (6 mm x 30 mm) and an RVC electrode were pierced through the septa. The electrodes were submerged to a depth of 5 mm into the solution. The reaction was removed from the glove box placed under N_2. A cell potential of 6 V was applied at room temperature for 4 equiv. e- and the solution was vigorously stirred (700 rpm). Following electrolysis, a basic workup was performed with 1M NaOH. The reaction mixture was then concentrated and loaded onto silica and purified by flash column chromatography. The conditions for chromatography and other data that are specific to each compound are given below.

General Procedure for Metal-Catalyzed Decarboxylative Addition Reactions

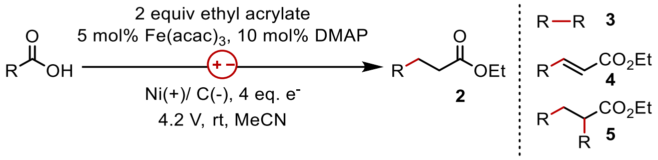

In a nitrogen filled glove box, a 12 mL reaction vial was charged with a stir bar, decanoic acid (129 mg, 0.750 mmol), 4-dimethylaminopyridine (9 mg, 70 µmol), $Fe(acac)_3$ (13 mg, 38 µmol) and ethyl acrylate (150. mg, 1.50 mmol) and MeCN (3 mL). The reaction vial was sealed with a septa-lined cap. A Ni foam electrode (6 mm x 30 mm) and an RVC electrode (6 mm x 30 mm) were pierced through the septa and were submerged to a depth of 5 mm into the solution. The reaction was removed from the glove box placed under N_2. An oxidative current was then applied to the Ni foam electrode (4.2 V, 4 equiv e-) at 25 °C and vigorously stirred (700 rpm). Following electrolysis, a basic workup was performed with 1M NaOH. The reaction mixture was then concentrated and loaded onto silica and purified by flash column chromatography. The conditions for chromatography and other data that are specific to each compound are given below.

Additional Supporting Electrolytes Tested

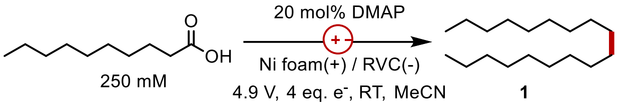

entry	deviation from standard	% yield
1	50 mM KPF_6	0
2	250 mM KPF_6	0
3	100 mM $LiClO_4$	0
4	100 mM $LiBF_4$	0
5	100 mM NBu_4Br	0
6	100 mM NBu_4PF_6	0
7	50 mM NH_4PF_6	0
8	100 mM NH_4PF_6	0
9	100 mM NH_4BF_4	0
10	100 mM $NaBF_4$	0
11	100 mM NEt_4ClO_4	0
12	100 mM TBACl	0

Supplementary Figure 1. List of additional supporting electrolytes tested with Kolbe homocoupling conditions. The yields are reported using GC analysis with dodecane as an internal standard.

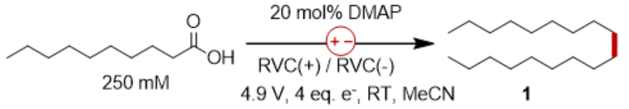

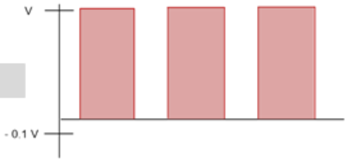

entry	duty cycle	on time	off time	% yield
1	70%	21 s	9 s	5
2	30%	9 s	21 s	16
3	70%	7 s	3 s	6
4	70%	3 s	7 s	8
5	67%	2 s	1 s	15
6	33%	1 s	2 s	14
7	50%	10 s	10 s	9
8	67%	20 s	10 s	11
9	75%	30 s	10 s	8
10	50%	1 min	1 min	10
11	83%	5 min	1 min	21

Supplementary Figure 2. Slow pulsing conditions tested with Kolbe homocoupling conditions. The yields are reported using GC analysis with dodecane as an internal standard.

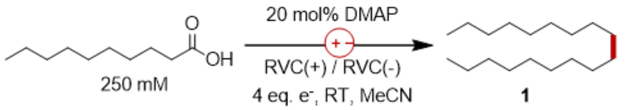

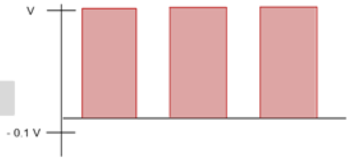

entry	voltage	duty cycle	frequency	% yield
1	4 V	67%	1.6 Hz	33
2	4 V	65%	10 Hz	31
3	4 V	67%	24 Hz	35
4	4 V	67%	73 Hz	25
5	4 V	67%	96 Hz	24
6	10 V	62%	5 Hz	55
7	10 V	62%	21 Hz	50
8	10 V	67%	48 Hz	46
9	10 V	62%	5 Hz	51

Supplementary Figure 3. Rapid pulsing conditions tested with Kolbe homocoupling conditions. The yields are reported using GC analysis with dodecane as an internal standard.

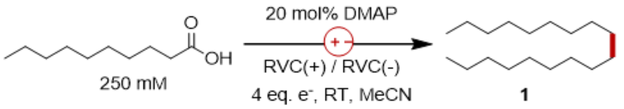

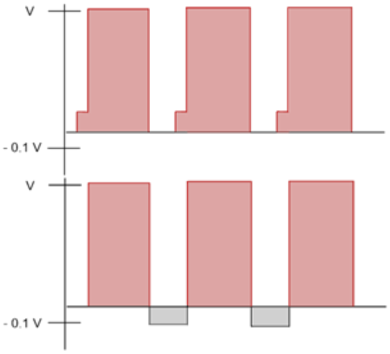

entry	initial charge	voltage	time on	time off	% yield
1	0.5 V (10 s)	4.9 V	30 s	10 s	6
2	0.5 V (30 s)	4.9 V	30 s	10 s	10
3	-0.5 V (10 s)	4.9 V	10 s	10 s	13
4	-0.5 V (10 s)	4.9 V	20 s	10 s	18
5	-0.5 V (10 s)	4.9 V	30 s	10 s	15

Supplementary Figure 4. Initial pulse and polarity switch pulsing conditions tested with Kolbe homocoupling conditions. The yields are reported using GC analysis with dodecane as an internal standard.

Classes of Tested Metals

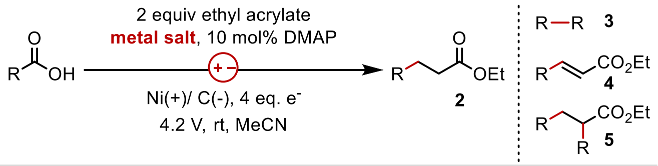

entry	deviation from standard	% yield	ratio 2 : (3+4+5)
1	5 mol% $NiBr_2$	0	-
2	5 mol% $NiCl_2$	0	-
3	5 mol% $MnCl_2$	0	-
4	5 mol% $CuBr_2$	0	-
5	5 mol% $CoBr_2$	0	-
6	5 mol% $FeCl_3$	0	-
7	5 mol% $Fe(Cl)_2(H_2O)_4$	0	-
8	5 mol% $Fe(SO_4)(H_2O)_7$	0	-
9	5 mol% $Fe(NO_3)_3(H_2O)_9$	0	-
10	5 mol% $Ni(NO_3)_2(H_2O)_6$	0	-
11	5 mol% $Fe(OAc)_2$ + 5 mol% BPI	0	-
12	5 mol% $Co(OAc)_2$	43	0.1 : 1
13	5 mol% Ag(OAc)	0	-
14	5 mol% $Ag(NO_3)$	0	-
15	5 mol% Ag_2CO_3	0	-

Supplementary Figure 5. List of tested metal salts with yields reported using GC analysis with dodecane as an internal standard.

Procedure for Electrode Preparation:

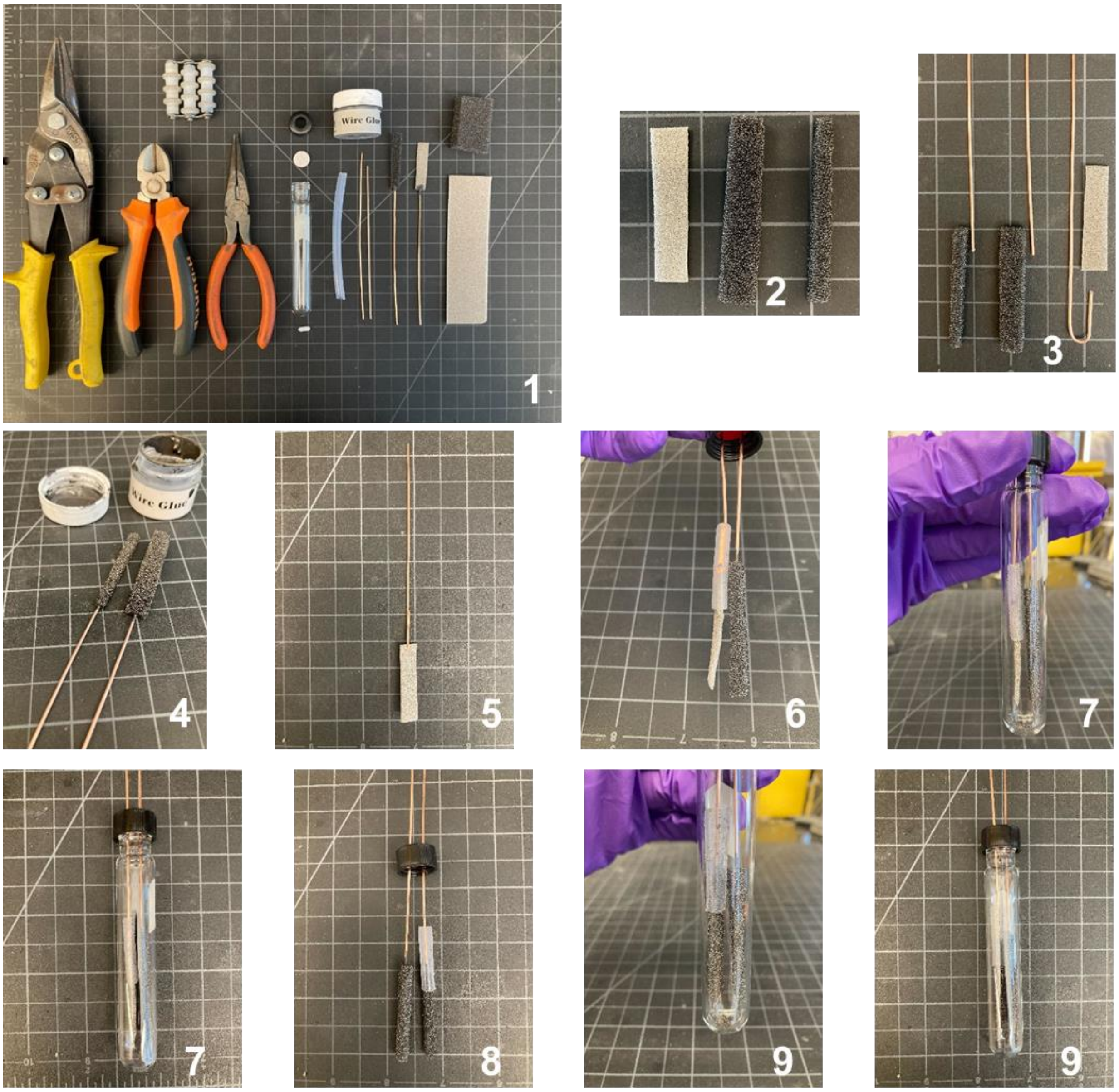

1. Materials required: 12 mL threaded reaction test tube, PTFE septa, threaded test tube cap, copper wire (18 ga), nickel foam, RVC, PTFE tubing (3/16” ID, 1/4” OD, 1/32” WT), pliers, wire glue, wire cutters, 10x3 mm stir bar, wire straighteners, and tin snips.

2. Use tin snips to cut the nickel foam into 6 mm x 32 mm strips and a razor to cut the RVC into 6 mm x 32 mm strips. Use a small cork borer (3/16 inch) to cut the cylindrical RVC electrode.

3. Use the wire cutters and wire straightener to cut the Cu wire and straighten the Cu leads.

4. Secure the RVC to the copper wire with wire glue and pierce the Cu wire through the RVC.

5. Secure the nickel foam to the copper wire. For the nickel foam cathode, the copper wire was threaded through the hole and the copper was folded back on itself to clamp the nickel foam in place.

6. On the Ni foam anode, a segment of PTFE tubing was cut and was placed over the Ni foam -copper connection – to prevent the electrodes from touching. Copper wire from the nickel foam and RVC electrodes were pushed through PTFE septa and electrodes were positioned parallel to each other to prevent contact.

7. Ni-foam and RVC electrodes were slid into the test tube, inserted until a 1 cm gap remains at the bottom of the test tube. The septa was then secured with the threaded cap.

8. On the circular RVC anode, a segment of PTFE tubing was cut and was placed over the RVC-copper connection – to prevent the electrodes from touching. Copper wire from the RVC electrodes were pushed through PTFE septa and electrodes were positioned parallel to each other to prevent contact.

9. RVC electrodes were slid into the test tube, inserted until a 1 cm gap remains at the bottom of the test tube. The septa was then secured with the threaded cap.

Procedure for Electrochemical Impedance Spectroscopy (EIS)

EIS measurements were performed in 3-neck reaction flask with a Ni-foam anode and Ni-foam cathode under the standard conditions for decarboxylative homocoupling (see general procedure for electrochemical dimerization). Electrodes were submerged in solution and the reference wire and counter wire were connected to the counter electrode. EIS measurements were acquired at an applied voltage of 2 V with an overlaid voltage perturbation of ±200 mV from a high frequency of 1000 kHz to a low frequency of 0.5 Hz. The Nyquist plots were modeled according to a modified Randles circuit with a Warburg element to estimate solution resistances for carboxylic acid solutions.

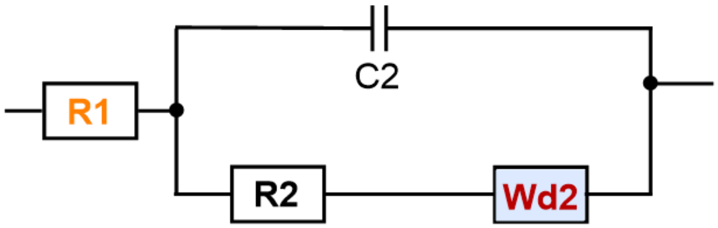

Circuit used for calculation in Bio-Logic software: R1+C2/(R2+Wd2)

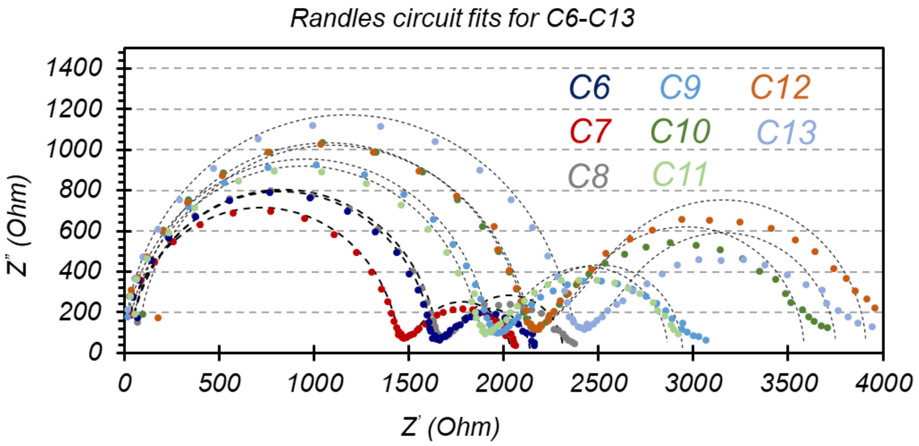

	6	7	8	9	10	11	12	13
R1 (Ohm)	27.4	3.976	31.38	1.845	33.3	14.6	66.21	0.1276
C2 (F)	8.008E-10	0.771	8.237E-10	7.8E-10	7.765E-10	7.63E-10	7.944E-10	7.66E-10
R2 (Ohm)	1587	1427	1603	1903	2060	1833	2034	2336
Rd2 (Ohm)	524.7	604.4	679.7	1038	1489	1016	1805	1419

Supplementary Figure 6. Illustration of Randles circuit that was fit on the pre-electrolysis EIS spectra (top). EIS spectra of Ni-foam electrodes submerged in C6 – C13 solution. The fits for each Nyquist plot are replicated with the black dashed traces. Values of the resistors, capacitors and Warburg elements of the Randles circuit for each carboxylic acid (bottom).

Circuit used in all trials and additional EIS studies:

Circuit used for calculation in Bio-Logic software: L1+R1+C1/(R2+Q3/R3)

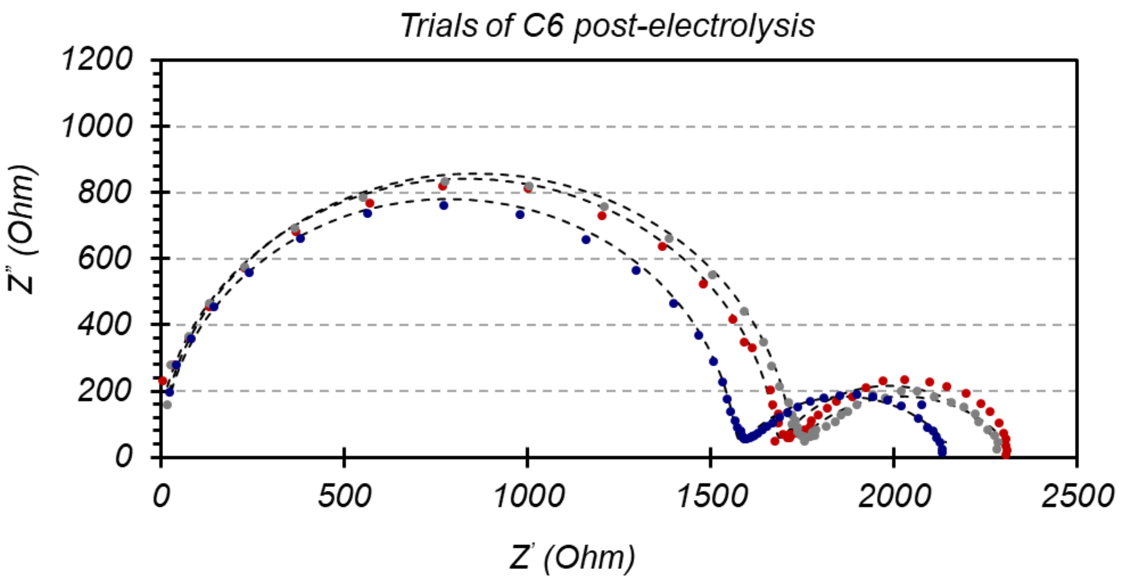

	R1 (Ohm)	R2 (Ohm)	R3 (Ohm)	L1 (H)	C1 (F)	a3	Q3 (F.s^(a - 1))
trial 1	1.953	1562	605.9	4.306E-07	7.668E-10	0.6924	0.00004311
trial 2	8.265	1684	622.8	7.697E-07	7.471E-10	0.7704	0.00001886
trial 3	2.15E-11	1716	620.3	9.729E-69	7.962E-10	0.6807	0.00004306
average	3.406	1654	616.333333	4.001E-07	7.7E-10	0.7145	0.00003501

Supplementary Figure 7. Nyquist plots from EIS and modeled fit (dashed traces) of three trials of C6 solution after 30 minutes of electrolysis.

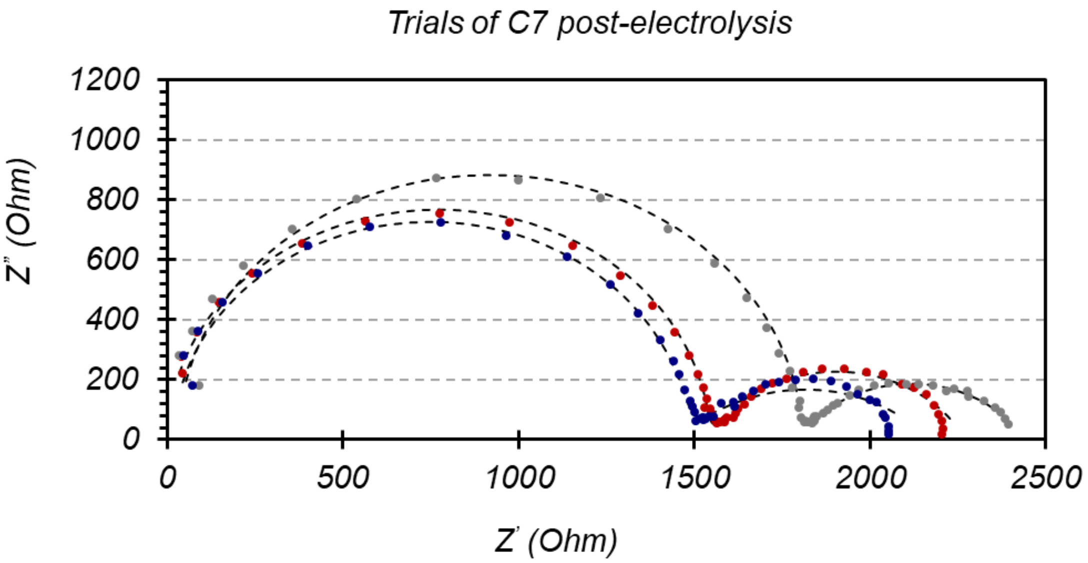

	R1 (Ohm)	R2 (Ohm)	R3 (Ohm)	L1 (H)	C1 (F)	a3	Q3 (F.s^(a - 1))
trial 1	14.73	1455	697.2	2.214E-06	7.578E-10	0.5696	0.00005671
trial 2	7.955	1531	732.8	-3.44E-06	7.798E-10	0.7066	0.000036
trial 3	31.36	1768	638.3	4.349E-07	7.91E-10	0.6745	0.00005068
average	18.015	1584.66667	689.433333	-2.65E-07	7.762E-10	0.650233	4.77967E-05

Supplementary Figure 8. Nyquist plots from EIS and modeled fit (dashed traces) of three trials of C7 solution after 30 minutes of electrolysis.

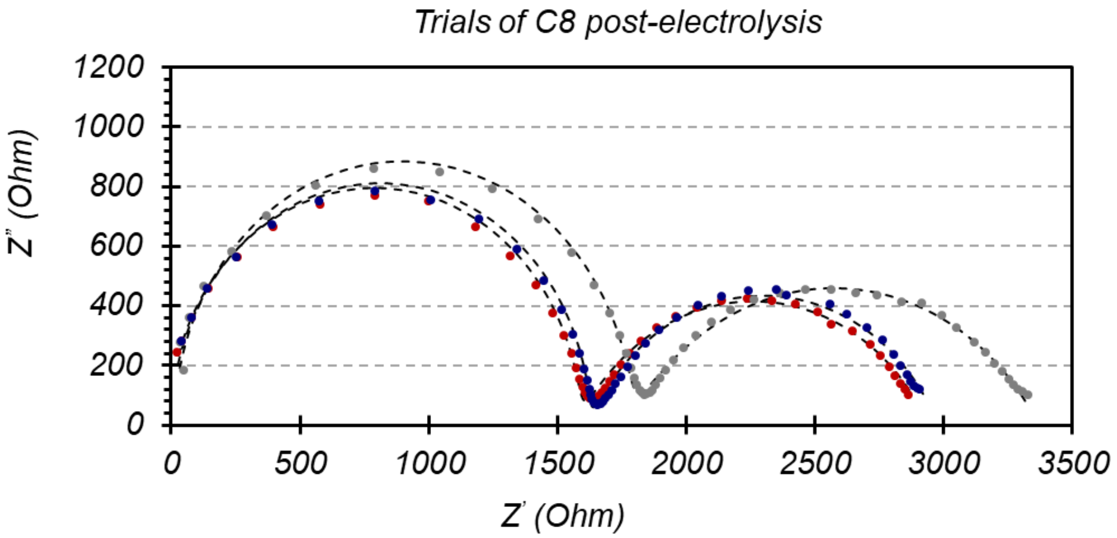

	R1 (Ohm)	R2 (Ohm)	R3 (Ohm)	L1 (H)	C1 (F)	a3	Q3 (F.s^(a - 1))
trial 1	5.09E-14	1623	1355	2.088E-165	7.598E-10	0.7252	0.00002747
trial 2	0.2648	1582	1357	0.000004632	7.65E-10	0.6985	0.00001967
trial 3	8.37E+00	1771	1588	0.000002697	7.531E-10	0.6807	0.00001956
average	2.87926667	1658.66667	1433.33333	0.000002443	7.593E-10	0.701467	2.22333E-05

Supplementary Figure 9. Nyquist plots from EIS and modeled fit (dashed traces) of three trials of C8 solution after 30 minutes of electrolysis.

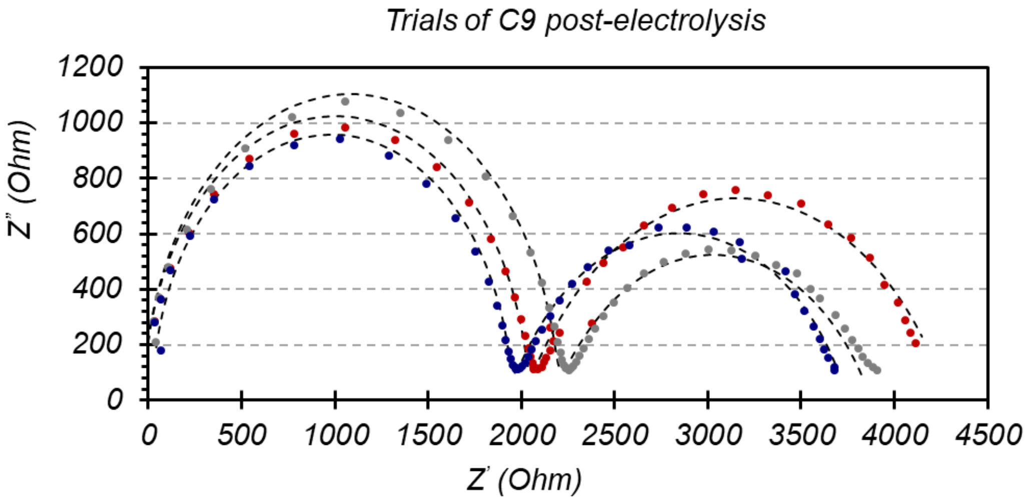

	R1 (Ohm)	R2 (Ohm)	R3 (Ohm)	L1 (H)	C1 (F)	a3	Q3 (F.s^(a - 1))
trial 1	19.35	1919	1812	0.000002272	7.662E-10	0.7465	0.00001255
trial 2	25.5	2051	2258	0.000001316	7.243E-10	0.7293	0.00001447
trial 3	24.01	2209	1691	0.000001853	7.19E-10	0.7063	0.00001804
average	22.9533333	2059.66667	1920.33333	1.81367E-06	7.365E-10	0.727367	0.00001502

Supplementary Figure 10. Nyquist plots from EIS and modeled fit (dashed traces) of three trials of C9 solution after 30 minutes of electrolysis.

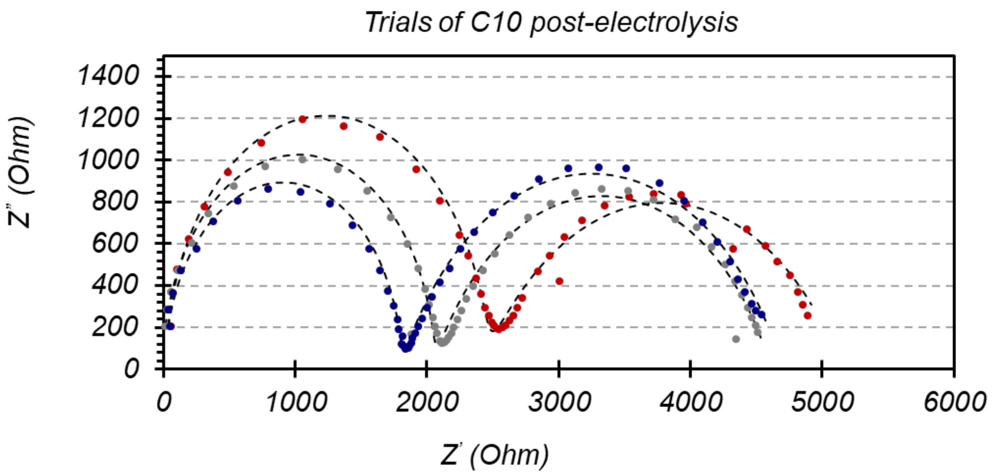

	R1 (Ohm)	R2 (Ohm)	R3 (Ohm)	L1 (H)	C1 (F)	a3	Q3 (F.s^(a - 1))
trial 1	14.22	1788	2897	5.31E-10	7.578E-10	0.7301	0.00001602
trial 2	19.24	2423	2702	2.968E-07	7.637E-10	0.6768	0.0000111
trial 3	4.168	2054	2563	3.843E-07	7.458E-10	0.7309	0.00001256
average	12.5426667	2088.33333	2720.66667	2.2721E-07	7.558E-10	0.7126	1.32267E-05

Supplementary Figure 11. Nyquist plots from EIS and modeled fit (dashed traces) of three trials of C10 solution after 30 minutes of electrolysis.

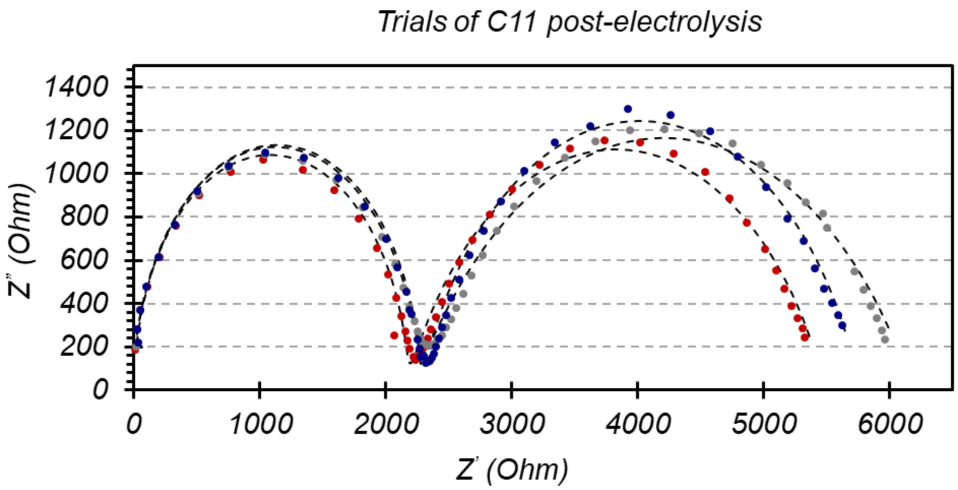

	R1 (Ohm)	R2 (Ohm)	R3 (Ohm)	L1 (H)	C1 (F)	a3	Q3 (F.s^(a - 1))
trial 1	7.824	2264	3496	0.000001969	7.631E-10	0.7873	0.00000938
trial 2	7.155	2178	3320	0.000003317	7.379E-10	0.7514	0.00000941
trial 3	1.669	2245	3938	8.275E-07	7.499E-10	0.6799	0.0000105
average	5.54933333	2229	3584.66667	2.03783E-06	7.503E-10	0.739533	9.76333E-06

Supplementary Figure 12. Nyquist plots from EIS and modeled fit (dashed traces) of three trials of C11 solution after 30 minutes of electrolysis.

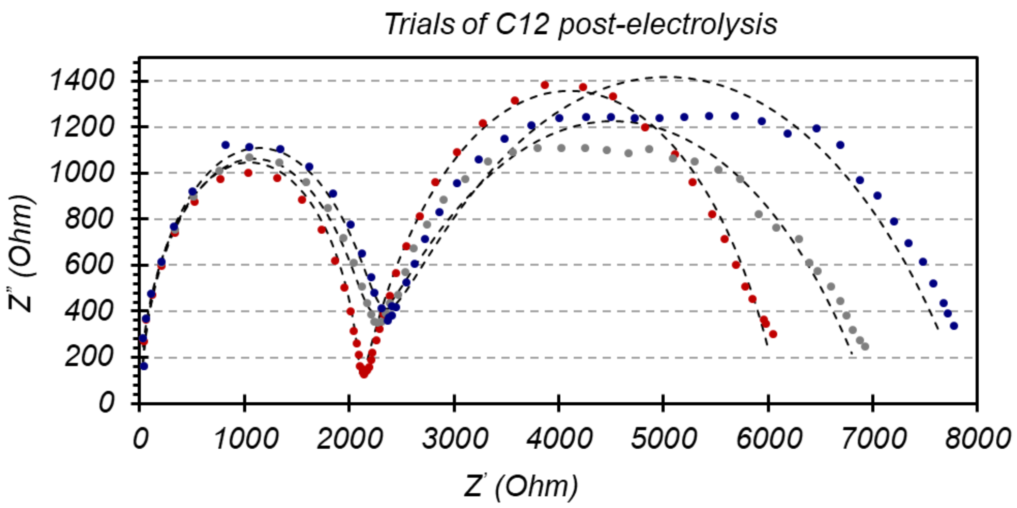

	R1 (Ohm)	R2 (Ohm)	R3 (Ohm)	L1 (H)	C1 (F)	a3	Q3 (F.s^(a - 1))
trial 1	6.479	2186	5708	0.000007397	7.229E-10	0.5854	0.000005943
trial 2	10.56	2085	4018	0.000007581	7.879E-10	0.7565	0.00001019
trial 3	6.402	2096	4875	0.000006993	7.3E-10	0.5912	0.000005807
average	7.81366667	2122.33333	4867	7.32367E-06	7.469E-10	0.644367	7.31333E-06

Supplementary Figure 13. Nyquist plots from EIS and modeled fit (dashed traces) of three trials of C12 solution after 30 minutes of electrolysis.

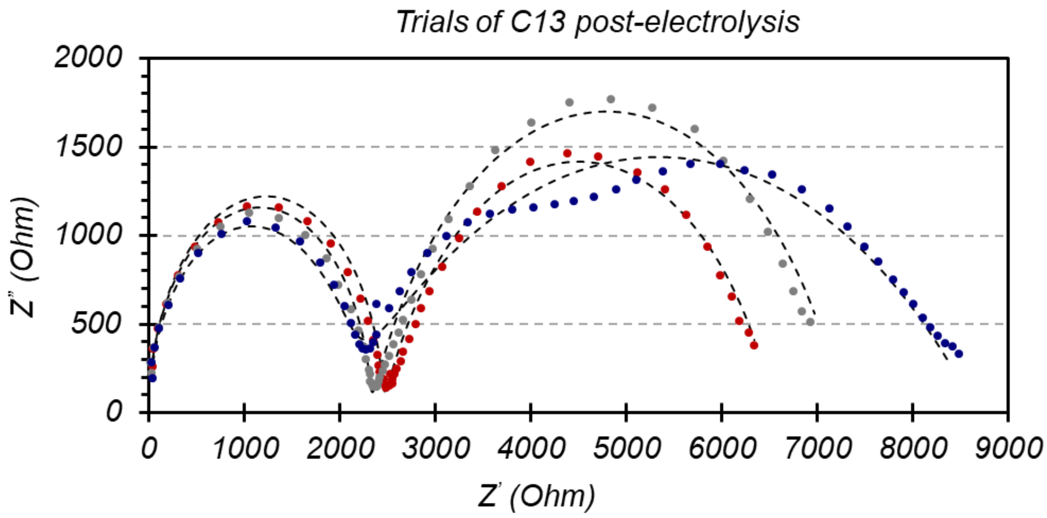

	R1 (Ohm)	R2 (Ohm)	R3 (Ohm)	L1 (H)	C1 (F)	a3	Q3 (F.s^(a - 1))
**trial 1**	6.072	2042	6618	0.000002067	7.376E-10	0.5227	0.00001017
**trial 2**	21.5	2432	4094	0.00001169	8.087E-10	0.7707	0.00001086
**trial 3**	7.255	2315	4944	0.000002378	7.595E-10	0.767	0.00001038
**average**	11.609	2263	5218.66667	5.37833E-06	7.686E-10	0.6868	0.00001047

Supplementary Figure 14. Nyquist plots from EIS and modeled fit (dashed traces) of three trials of C13 solution after 30 minutes of electrolysis.

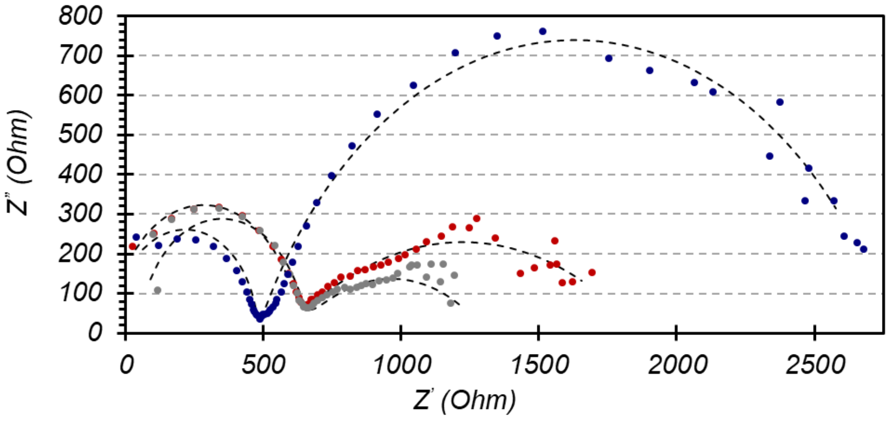

Supplementary Figure 15. EIS spectrum of a Ni-foam anode that was electrolyzed in C12 solution for 30 min (blue trace) and then moved to a C6 solution. After 10 minutes with rapid stirring additional EIS was taken (red trace), then again after an additional 30 minutes (grey trace). This demonstrates that the surface layer is reversible and the C12 layer can be replace by the C6 acid at the electrode surface.

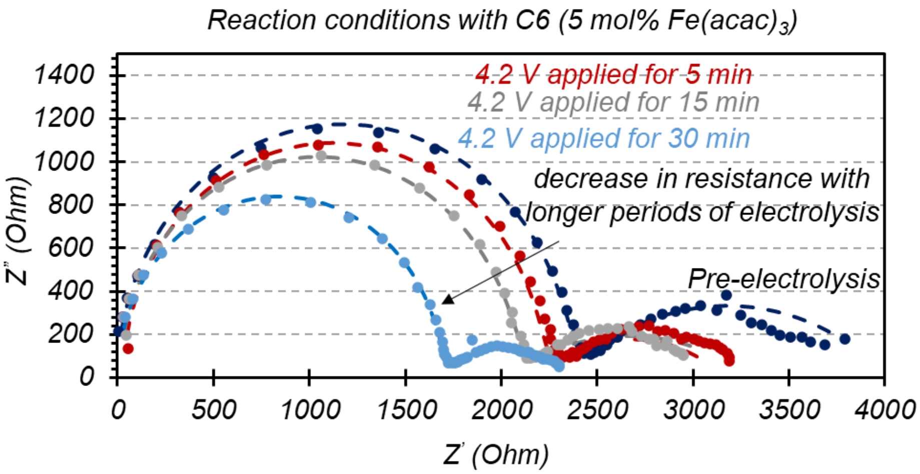

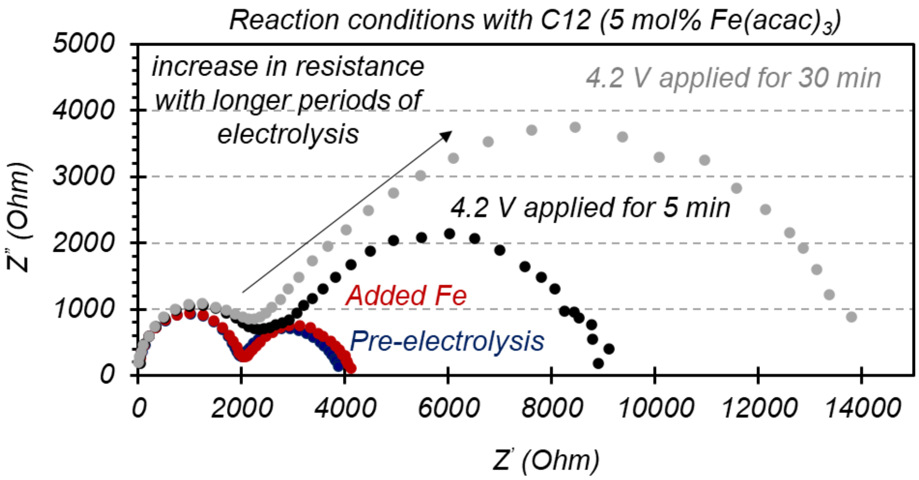

Supplementary Figure 16. EIS spectra with added $Fe(acac)_3$. SAMs are still able to form in the presence of Fe with the long chain substrates, but not with the short chain substates.

General Procedure for Divided Cell Reaction

In a nitrogen filled glove box, each chamber of an H-cell was charged with a stir bar, decanoic acid (194 mg, 1.13 mmol), 4-dimethylaminopyridine (14 mg, 11 mmol), $Fe(acac)_3$ (20 mg, 57 µmol) and ethyl acrylate (225 mg, 2.25 mmol) and MeCN (4.5 mL). The reaction vial was sealed with a septa-lined cap. A Ni foam electrode (6 mm x 30 mm) and an RVC electrode (6 mm x 30 mm) were pierced through the septa and were submerged to a depth of 5 mm into the solution. An oxidative current was then applied to the Ni foam electrode (1 mA, 24 hr, 24 mAhr) at 25 °C and vigorously stirred (700 rpm). Following electrolysis, a basic workup was performed with 1M NaOH and analyzed by GC.

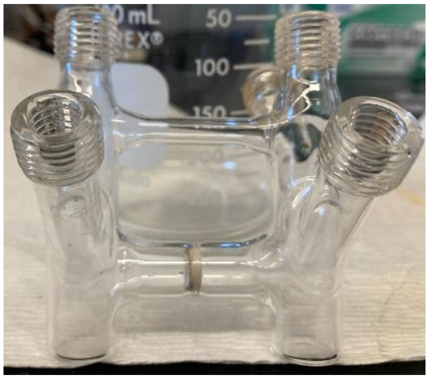

	Anodic Chamber	Cathodic Chamber
Target Product **2**	<5%	0%
Side Products **3-5**	12%	0%

Supplementary Figure 17. (Top) Picture of the H-cell used. (Bottom) GC yield obtained from the anodic chamber and cathodic chamber.

Substrate Scope Limitations (Supplementary Figure 18-20):

Short Chain Carboxylic Acids

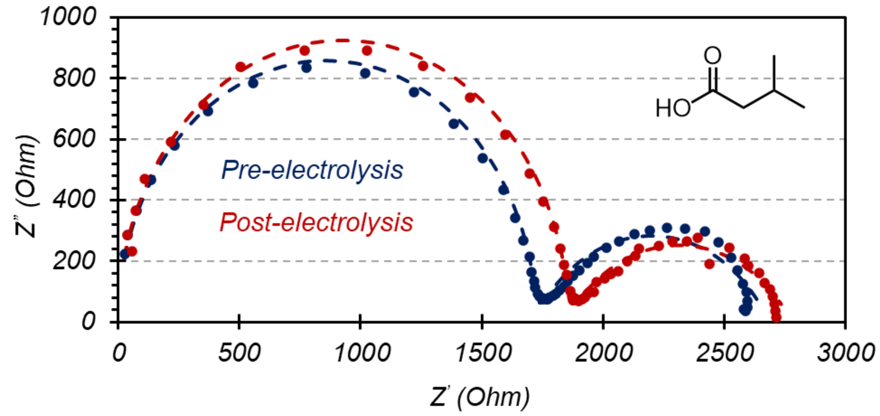

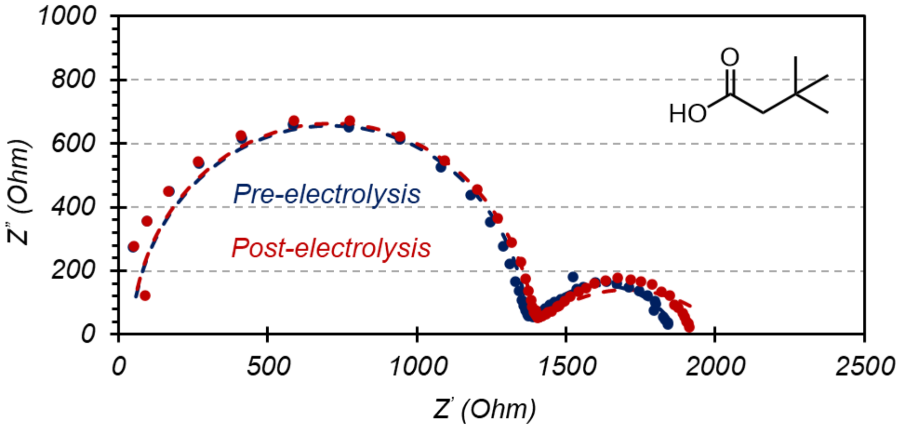

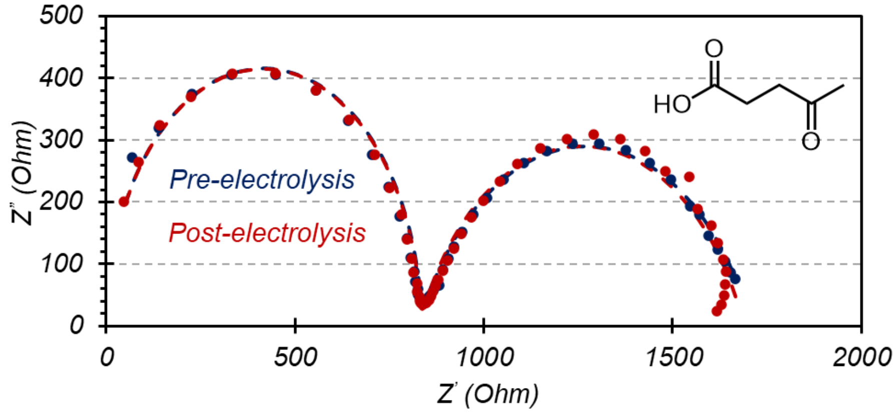

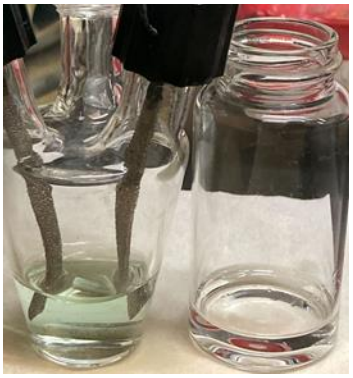

Supplementary Figure 18. EIS spectra of various short chain acids before (blue trace) and after electrolysis (red, 4.2 V for 30 min). A picture of the short chain solution before (right) and after (left) electrolysis. The smaller substrates do not form SAMs at the electrode surface. This is demonstrated by EIS spectra that are identical before and after electrolysis. When SAMs are not formed, the Ni electrode is oxidized, and Ni is leached into the solution leading to a green solution (left).

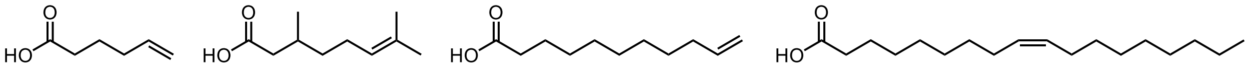

Supplementary Figure 19. Alkenes can be oxidized at the anodic surface and passivate the electrode surface. The current with all alkenyl substrates decreased significantly after ~30 min of electrolysis, indicating that the electrode was passivated.

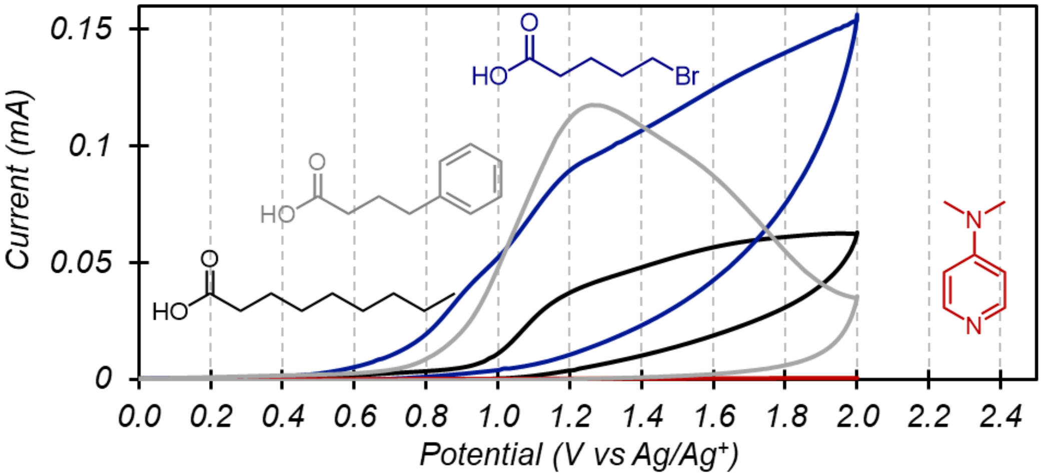

Supplementary Figure 20. The reported electrochemical decarboxylation proceeds at lower voltages typically utilized for Kolbe reactions, however, <4 V is still too high for common oxidatively sensitive functional groups. Arenes and halides on the carboxylic acid are oxidized preferentially to the alkyl carboxylic acid.

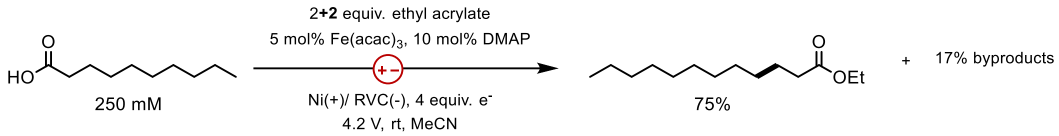

Supplementary Figure 21. Portions of addition acrylate can be added during the reaction (~2 e^- equiv.) to increase the yield for the monofunctionalized product.

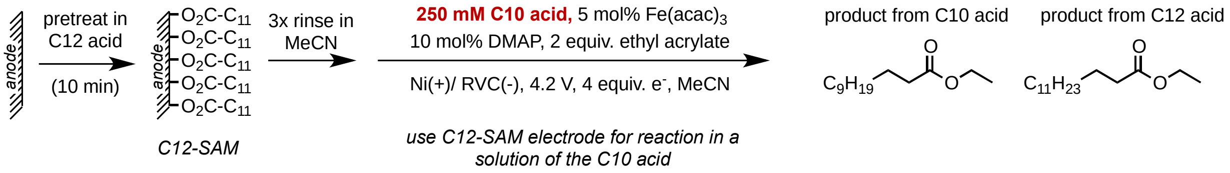

Timepoint	Monofunctionalized (C10)	Monofunctionalized (C12)
5 min	<1%	2%
3 hr	10%	8%
12hr (Tf)	65%	12%

Supplementary Figure 22. Competition experiment with a pre-treated C12 anode. This experiment demonstrated that C12 is adsorbed onto the Ni-foam surface and undergo decarboxylation to form product even with an excess of C10 acid.

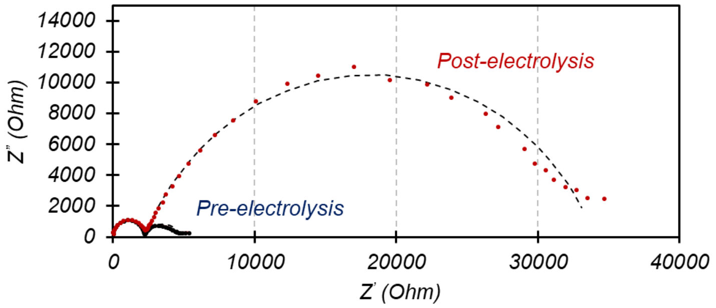

Supplementary Figure 23. EIS spectra before and after electrolysis with Pt electrodes demonstrating that a large amount of resistance is observed with Pt electrodes. This results in a low current sustained throughout the reaction.

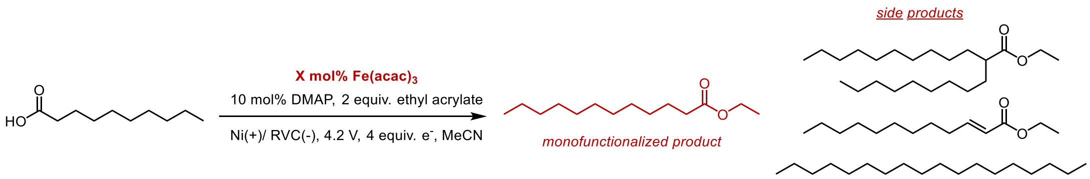

[Carboxylic Acid]	$Fe(acac)_3$ loading	Product Yield	Side Product Yield	Selectivity

250 mM	5 mol%	68%	12%	85%
250 mM	10 mol%	53%	3%	95%
500 mM	2.5 mol%	51%	16%	76%

Supplementary Figure 24. Comparative study evaluating the influence of $Fe(acac)_3$ loading and carboxylic acid concentration on selectivity. The catalyst loading of 5 mol% is primarily a concentration effect that ensures efficient radical capture. Reactions with less Fe are less selective because the radical capture is slower. As an example, if we double the concentration of substrate, we can maintain the same concentration of Fe and achieve similar selectivities.

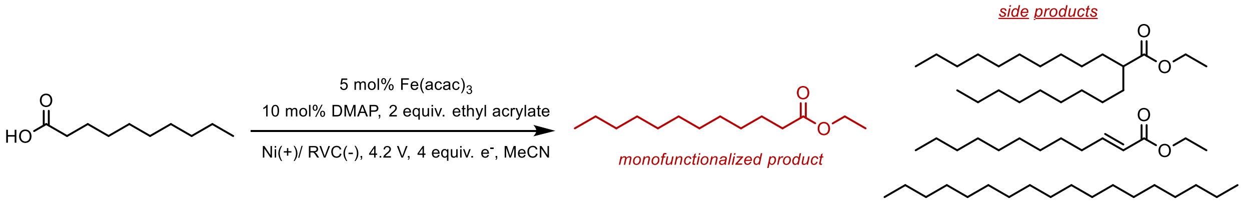

[Carboxylic Acid]	*Product Yield*	*Side Product Yield*	*Total Yield*	*Selectivity*
250 mM	*62%*	*14%*	*76%*	*82%*

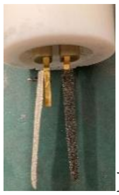
Ni foam (+)/ RVC (-)

Supplementary Figure 25. The reaction was performed with an Ika Electrasyn 2.0 and products were formed in similar yields and selectivities as those observed with our standard cells. The electrode setup is pictured to show the setup of the Ni foam anode and RVC cathode.

Characterization of Isolated Products

6 – Ethyl decanoate

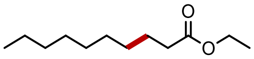
The general procedure described above was applied to the reaction of octanoic acid (108 mg, 0.75 mmol) with ethyl acrylate (150 mg, 1.5 mmol). The reaction was removed from the glove box placed under N_2. An oxidative current was then applied to the Ni foam electrode (4.2 V, 4 equiv e-) at 25 °C and vigorously stirred (700 rpm). The crude product was isolated by column chromatography with 100% hexanes to afford the title compound as a clear oil (77 mg, 51%). Characterization data match those of previously reported literature.[1]

^{1}H NMR (400 MHz, $CDCl_3$) δ 4.15 – 4.10 (q, J = 7.1 Hz, 2H), 2.30 – 2.26 (m, 2H), 1.63 – 1.60 (m, 2H), 1.32 – 1.23 (m, 15H), 0.89 – 0.86 (m, 3H).

^{13}C NMR (101 MHz, $CDCl_3$) δ 174.1, 60.3, 34.6, 32.0, 29.6, 29.4 (2C), 29.3, 25.2, 22.8, 14.4, 14.3.

7 – Ethyl 6-methyldecanoate

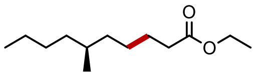
The general procedure described for metal-catalyzed decarboxylative additions was applied to the reaction of 4-methyloctanoic acid (119 mg, 0.75 mmol) with ethyl acrylate (150 mg, 1.5 mmol). The reaction was removed from the glove box placed under N_2. An oxidative current was then applied to the Ni foam electrode (4.2 V, 4 equiv e-) at 25 °C and vigorously stirred (700 rpm). The crude product was isolated by column chromatography with 100% hexanes to afford the title compound as a clear oil (68 mg, 42%). See copies of spectra below.

^{1}H NMR: (400 MHz, Chloroform-*d*) δ 4.15 – 4.10 (q, *J* = 7.1 Hz, 2H), 2.31 – 2.27 (t, *J* = 7.5 Hz, 2H), 1.62 – 1.56 (m, 2H), 1.30 – 1.21 (m, 12H), 1.12 – 1.07 (m, 2H), 0.90 – 0.83 (m, 6H).

^{13}C NMR (101 MHz, $CDCl_3$) δ 174.1, 60.3, 36.8 (2C), 34.6, 32.7, 29.44, 26.8, 25.5, 23.2, 19.8, 14.4, 14.3.

IR (neat) ν_{max} (cm^{-1}): 2959, 2925, 2864, 2857, 1738, 1467, 1371, 1162, 1110, 1029.

8 – Ethyl dodecanoate

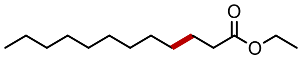
The general procedure described above was applied to the reaction of decanoic acid (129 mg, 0.750 mmol) with ethyl acrylate (150. mg, 1.50 mmol). The reaction was removed from the glove box placed under N_2. An oxidative current was then applied to the Ni foam electrode (4.2 V, 4 equiv e-) at 25 °C and vigorously stirred (700 rpm). The crude product was isolated by column chromatography with 100% hexanes to afford the title compound as a clear oil (106 mg, 62%). Characterization data match those of previously reported literature.[2]

^{1}H NMR: (400 MHz, $CDCl_3$) δ 4.14 – 4.09 (q, *J* = 7.1 Hz, 2H), 2.29 – 2.26 (m, 2H), 1.65 – 1.57 (m, 2H), 1.32 – 1.22 (m, 19H), 0.89 – 0.85 (m, 3H).

^{13}C NMR: (101 MHz, $CDCl_3$) δ 173.9, 60.1, 34.4, 31.9, 29.6 (2C), 29.5, 29.3, 29.3, 29.2, 25.0, 22.7, 14.3, 14.1.

9 – Dodecanenitrile

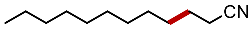
The general procedure described above was applied to the reaction of decanoic acid (129 mg, 0.750 mmol) with acrylonitrile (80 mg, 1.5 mmol). The reaction was removed from the glove box placed under N_2. An oxidative current was then applied to the Ni foam electrode (4.2 V, 4 equiv e-) at 25 °C and vigorously stirred (700 rpm). The crude product was isolated by column chromatography with 100% hexanes to afford the title compound as a clear oil (58 mg, 43%). Characterization data match those of previously reported literature.[3]

^{1}H NMR: (400 MHz, $CDCl_3$) δ 2.34 – 2.30 (t, *J* = 7.1 Hz, 2H), 1.68 – 1.61 (m, 2H), 1.46 – 1.40 (m, 2H), 1.34 – 1.22 (m, 14H), 0.89 – 0.85 (m, 3H).

^{13}C NMR: (101 MHz, $CDCl_3$) δ 112.0, 32.0, 29.7, 29.6, 29.4, 29.4, 28.9, 28.8, 25.5, 22.8, 17.2, 14.2.

10 – Tridecan-2-one

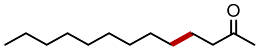
The general procedure described above was applied to the reaction of decanoic acid (129 mg, 0.75 mmol) with methyl vinyl ketone (105 mg, 1.50 mmol). The reaction was removed from the glove box placed under N_2. An oxidative current was then applied to the Ni foam electrode (4.2 V, 4 equiv e-) at 25 °C and vigorously stirred (700 rpm). The crude product was isolated by column chromatography with 100% hexanes to afford the title compound as a light-yellow oil (60 mg, 40%). Characterization data match those of previously reported literature.[4]

^{1}H NMR: (400 MHz, $CDCl_3$) δ 2.42 – 2.38 (t, *J* = 7.1 Hz, 2H), 2.11 (s, 3H), 1.57 – 1.53 (m, 2H), 1.32 – 1.20 (m, 16H), 0.88 – 0.84 (m, 3H).
^{13}C NMR: (101 MHz, $CDCl_3$) δ 209.6, 44.0, 32.0, 30.0, 29.7, 29.6, 29.5, 29.5, 29.3, 24.0, 22.8, 22.8, 14.3.

11 – Ethyl tetradecanoate

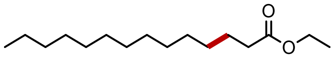

The general procedure described above was applied to the reaction of lauric acid (150 mg, 0.75 mmol) with ethyl acetate (150 mg, 1.5 mmol). The reaction was removed from the glove box placed under N_2. An oxidative current was then applied to the Ni foam electrode (4.2 V, 4 equiv e-) at 25 °C and vigorously stirred (700 rpm). The crude product was isolated by column chromatography with 100% hexanes to afford the title compound as a clear oil (92 mg, 48%). Characterization data match those of previously reported literature.[5]
^{1}H NMR (400 MHz, $CDCl_3$) δ 4.15 – 4.10 (q, *J* = 7.1 Hz, 2H), 2.30 – 2.25 (m, 2H), 1.63 – 1.59 (m, 2H) 1.59, 1.32 – 1.23 (m, 23H), 0.90 – 0.85 (m, 3H).
^{13}C NMR: (101 MHz, $CDCl_3$) δ 174.1, 60.3, 34.6, 32.1, 29.8, 29.8 (2C), 29.6, 29.5, 29.4, 29.3, 25.2, 22.9, 22.8, 14.4, 14.3.

12 – Ethyl 3-cyclopropanoate

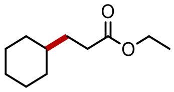

The general procedure described above was applied to the reaction of cyclohexyl carboxylic acid (96 mg, 0.75 mmol) with ethyl acetate (150 mg, 1.50 mmol). The reaction was removed from the glove box placed under N_2. An oxidative current was then applied to the Ni foam electrode (4.2 V, 4 equiv e-) at 25 °C and vigorously stirred (700 rpm). The crude product was isolated by column chromatography with 0.5% EtOAc in hexanes to afford the title compound as a clear oil (47 mg, 34%). Characterization data match those of previously reported literature.[6]
^{1}H NMR (400 MHz, $CDCl_3$) δ 4.14 – 4.09 (q, *J* = 7.1 Hz, 2H), 2.31 – 2.29 (m, 2H), 1.72 – 1.67 (m, 4H), 1.55 – 1.49 (m, 2H), 1.27 – 1.17 (m, 8H), 0.90 – 0.87 (m, 2H).
^{13}C NMR (101 MHz, CDCl3) δ 174.4, 60.3, 37.4, 33.1 (2C), 32.5, 32.1, 26.7, 26.4 (2C), 14.4.

13 – Ethyl icosanoate

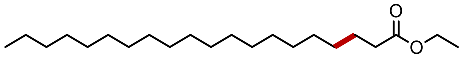

The general procedure described above was applied to the reaction of cyclohexyl carboxylic acid (96 mg, 0.75 mmol) with ethyl acetate (150 mg, 1.50 mmol). The reaction was removed from the glove box placed under N_2. An oxidative current was then applied to the Ni foam electrode (4.2 V, 4 equiv e-) at 25 °C and vigorously stirred (700 rpm). The crude product was isolated by column chromatography with 100% hexanes to afford the title compound as a clear oil (138 mg, 54%).
^{1}H NMR: (400 MHz, $CDCl_3$) δ 4.14 – 4.09 (q, *J* = 7.1 Hz, 2H), 2.32 – 2.28 (t, *J* = 7.6 Hz, 2H), 1.65 – 1.62 (m, 2H) 1.34 – 1.22 (m, 35H), 0.92 – 0.88 (m, 3H).
^{13}C NMR: (101 MHz, $CDCl_3$) δ 173.9, 60.1, 34.4, 31.9, 29.7(8C), 29.6, 29.5, 29.4 (2C), 29.3, 29.2, 25.0, 22.7, 14.26, 14.12.
IR (neat) ν_{max} (cm^{-1}): 2916, 2848, 1737, 1466, 1375, 1198, 1169, 1105, 1030, 720.

Supplementary References

1. Bian, T. *et al.* Oxidative Esterification of Aldehydes and Alcohols Catalyzed by Camphor-Based Imidazolium Salts. *Catal. Letters* **150**, 1812–1820 (2020).

2. Badgujar, K. C. & Bhanage, B. M. Immobilization of lipase on biocompatible co-polymer of polyvinyl alcohol and chitosan for synthesis of laurate compounds in supercritical carbon dioxide using response surface methodology. *Process Biochem.* **50**, 1224–1236 (2015).

3. Chatterjee, B., Jena, S., Chugh, V., Weyhermüller, T. & Werlé, C. A Molecular Iron-Based System for Divergent Bond Activation: Controlling the Reactivity of Aldehydes. *ACS Catal.* **11**, 7176–7185 (2021).

4. Huang, Z. *et al.* Oxidative Cleavage of Alkenes by O2with a Non-Heme Manganese Catalyst. *J. Am. Chem. Soc.* **143**, 10005–10013 (2021).

5. Cahiez, G., Chaboche, C., Duplais, C., Giulliani, A. & Moyeux, A. Cobalt-catalyzed cross-coupling reaction between functionalized primary and secondary alkyl halides and aliphatic Grignard reagents. *Adv. Synth. Catal.* **350**, 1484–1488 (2008).

6. Shukla, P., Hsu, Y. C. & Cheng, C. H. Cobalt-catalyzed reductive coupling of saturated alkyl halides with activated alkenes. *J. Org. Chem.* **71**, 655–658 (2006).
